# NO_2_ and Cancer Incidence in Saudi Arabia

**DOI:** 10.3390/ijerph10115844

**Published:** 2013-11-04

**Authors:** Khalid Al-Ahmadi, Ali Al-Zahrani

**Affiliations:** 1 King Abdulaziz City for Science and Technology, P.O. Box 6086, Riyadh 11442, Saudi Arabia; 2 King Faisal Specialist Hospital & Research Centre, P.O. Box 3354, Riyadh 11211, Saudi Arabia; E-Mail: alisaz@kfshrc.edu.sa

**Keywords:** cancer, NO_2_, Saudi Arabia, ordinary least square, geographically weighted regression, geographic information system

## Abstract

Air pollution exposure has been shown to be associated with an increased risk of specific cancers. This study investigated whether the number and incidence of the most common cancers in Saudi Arabia were associated with urban air pollution exposure, specifically NO_2_. Overall, high model goodness of fit (GOF) was observed in the Eastern, Riyadh and Makkah regions. The significant coefficients of determination (r^2^) were higher at the regional level (r^2^ = 0.32–0.71), weaker at the governorate level (r^2^ = 0.03–0.43), and declined slightly at the city level (r^2^ = 0.17–0.33), suggesting that an increased aggregated spatial level increased the explained variability and the model GOF. However, the low GOF at the lowest spatial level suggests that additional variation remains unexplained. At different spatial levels, associations between NO_2_ concentration and the most common cancers were marginally improved in geographically weighted regression (GWR) analysis, which explained both global and local heterogeneity and variations in cancer incidence. High coefficients of determination were observed between NO_2_ concentration and lung and breast cancer incidences, followed by prostate, bladder, cervical and ovarian cancers, confirming results from other studies. These results could be improved using individual explanatory variables such as environmental, demographic, behavioral, socio-economic, and genetic risk factors.

## 1. Introduction

A thorough understanding of the consequences of air pollutants on public health is essential for the progress of functioning policies to decrease the negative impact of ambient air pollution [1]. Mounting evidence indicates that exposure to air pollution might be associated with an increased risk of adverse health effects. An association has been reported between exposure to pollutants, such as particulate matter (PM), nitrogen dioxide (NO_2_) and ozone (O_3_), and increases in hospital admissions for cardiovascular and respiratory disease and mortality in Europe and the United Sates [2].

Several studies have found a relationship between the risk of developing cancer and exposure to air pollution [3,4,5], and many have concluded that long-term exposure to PM air pollution is positively associated with increased lung cancer mortality [6,7,8]. Nyberg *et al*. [4] used nitrogen oxide (NOx)/NO_2_ and SO_2_ as air pollution indicators from road traffic and heating and found that urban air pollution increased lung cancer risk. Based on the well-documented urban/rural difference in lung cancer incidence in Oslo, Nafstad *et al*. [5] found that the adjusted risk ratio for developing lung cancer was associated with NOx exposure between 1974 and 1978. Vineis *et al*. [9] assessed the relationship between air pollution (NO_2_, PM_10_, and SO_2_) and lung cancer in Europe. They found an association between lung cancer and NO_2_, while no obvious association was observed for other pollutants. In another recent study, an estimated 5%–7% of lung cancers in European non-smokers and ex-smokers could be attributed to exposure to high levels of air pollution, including NO_2_, or vicinity to heavy-traffic roads [10]. Evidence for an association between long-term exposure to air pollution and lung cancer is not limited to populations in Western countries. A study conducted by Katanoda *et al*. [11] demonstrated that long-term exposure to air pollution (PM_2.5_, SO_2_ and NO_2_) was related to the development of lung cancer and respiratory diseases in Japan. Raaschou-Nielsen *et al*. [12] found a relationship between NOx concentration and lung cancer risk and living within 50 m of a major road.

Although most studies have focused on the association between air pollution and lung cancer, there is evidence that air pollution is associated with an increased risk for other cancers. Castano-Vinyals *et al*. [13] reported small-to-moderate positive relationships between bladder cancer and a number of air pollution indicators. A trend analysis conducted in Taiwan demonstrated a significant relationship between increases in air pollution and risk of death from bladder cancer [14]. Crouse *et al*. [15] examined whether postmenopausal breast cancer was related to urban air pollution using NO_2_ as an indicator of air pollution. They found an approximately 25% increased risk of postmenopausal breast cancer for every 5 ppb increase in exposure to the ambient NO_2_ concentration. Raaschou-Nielsen *et al*. [16] investigated the association between traffic-related air pollution and risk for cancers other than lung cancer; they modeled the NOx concentration and traffic at the residence level as air pollution indicators from traffic. NOx at the residence level was considerably related to brain and cervical cancer risk. Rosenlund *et al*. [17] temporally analyzed all of the cancer cases that occurred in Stockholm County between 1985 and 1996 and suggested that long-term exposure to traffic-generated air pollutants such as NO_2_ increases the risk of cancer. Based on a follow-up evaluation that was conducted in 1999 and 2000 using annual average air pollution exposure data from 1991 to 2000, Kan and Gu [18] found significant associations between air pollutants (TSP, SO_2_ and NOx) and mortality from lung cancer in China. Using time-varying Cox proportional hazards models, Yorifuji *et al*. [19] provided support for the prevailing evidence that long-term exposure to traffic-related NO_2_ air pollution increases the risk of cardiopulmonary mortality as well as lung cancer mortality. In a Canadian study, Hystad *et al*. [20] developed spatiotemporal models to investigate lung cancer incidence in relation to long-term exposure to ambient air pollutants and found that lung cancer incidence increased most with NO_2_ and PM_2.5_ exposure.

However, other studies have reported a moderate, low or no evidence of association between the risk of adverse health effects and air pollution. Beelen *et al*. [21], for example, investigated the association between lung cancer incidence and air pollution using exposure to black smoke, NO_2_, SO_2_ and PM as well as traffic intensity variables as air pollution indicators. The relative risks were slightly below unity for the overall air pollution concentrations, while they were slightly elevated for the traffic variables.

Exposure to air pollution such as NO_2_ might be considered to be one environmental risk factor for cancer; however, cancer incidence rates are influenced by a combination of genetic, demographic, socio-economic and environmental risk factors [22,23,24,25,26,27,28,29,30,31,32]. Regrettably, there seems to be a lack of data on these covariates in Saudi Arabia, and thus these covariates could not be analyzed in the present study.

Most of the abovementioned studies used either logistic regression or classical global regression techniques, such as ordinary least square (OLS) regression, which presuppose that the relationship between cancer and air pollution is spatially invariant, homogeneous and stationary, *i.e.*, there are no local variations in the associations between the dependent and explanatory variables. The concept of stationarity is central in the analysis of spatial and temporal variations. A stationary process is a process that has similar properties at all locations in the area of interest. A stationary model has the same parameters at all locations, whereas a non-stationary model allows the parameters to vary locally [33]. Geographically weighted regression (GWR) is a local spatial statistical method used to examine spatial non-stationarity by allowing the associations between variables to vary from location to location [34]. GWR is a simple but powerful method for exploring non-stationary spatial relationships. It is a useful exploratory analytical tool that generates a set of location-specific parameter estimates that can be mapped and analyzed to provide information about spatial non-stationarity in the relationships between the predictors and the outcome variable [35]. The core principle underlying several local methods is the notion of spatial dependency: features close together in space tend to be more similar than features that are farther apart. This principle was termed the “First Law of Geography” by Tobler [36]. GWR is capable of extending the same principle to regression analysis [33].

The applications of GWR have grown rapidly in various fields, including sociology, health and demography [35]. GWR studies in health fields include the analysis of health and disease [37,38,39,40], health care delivery [41], the spatially varying relationships between immature mosquitoes and human population density [42] and gastric cancer in Taiwanese ethnic communities [43]. Mandal *et al*. [44] used OLS and GWR to examine whether breast cancer in females and prostate cancer in males were correlated at the county level in the United States using age-adjusted county-level average annual incidence rates for Caucasians. GWR revealed a more pronounced association than did OLS, and the parameter estimates computed for each county in the GWR model helped to determine that over 76% of the counties had a significant positive association between breast and prostate cancer. A more relevant study to the present research was conducted by Gilbert and Chakraborty [45], who stated that the spatial association between the cumulative cancer risk from exposure to hazardous air pollutants and explanatory variables such as race, ethnicity and socioeconomic status is not stationary throughout Florida’s census tracts. They found that conventional multivariate regression techniques such as OLS cannot reveal the local variations in these associations, whereas GWR allowed them to examine the spatial variation within the study area for each individual model coefficient.

Cancer incidence and mortality demonstrate non-stationary processes with regional variation and spatial drift, as they occur at different rates in different places. However, few studies in the literature have reported the use of GWR to assess the relationship between cancer incidence and tropospheric NO_2_. The present study aimed to investigate whether the number and incidence of the most common cancers in Saudi Arabia were significantly associated with exposure to urban air pollution (using NO_2_ as an indicator) using OLS and GWR in a Geographical Information System (GIS).

## 2. Materials and Methods

### 2.1. Cancer Data

Incidences of cancer were obtained from the Saudi Arabian Cancer Registry (SCR) [46]. The cancer dataset included data on diagnosed incidences of cancer in Saudi nationals from January 1998 to December 2004. A total of 45,532 cancer patients were diagnosed during this period. Many cancer indices have been devised to express the occurrence of cancer and other diseases in each zone. Three of the most common indices for cancer research are the crude incidence rate (CIR), the age-specific incidence rate (AIR) and the age-standardized incidence rate (ASR) [47,48]. In this study, the CIR for a particular cancer site in the human body is the total number of cases registered as a proportion of the total population. All rates were expressed as per 100,000 population. The CIR per 100,000 can readily be calculated by dividing the total number of cases of a particular cancer type by the population and multiplying the result by 100,000. Although age is a well-known covariate for cancer incidence, in the present study, the CIR was considered as opposed to an ASR. This decision was made because population data by age group are only available at the regional level, not at the governorate and city levels, and thus, ASRs could not be computed for governorates and cities. Furthermore, to ensure the consistency of the cancer rates for comparison across the three spatial levels, the CIR was most appropriate measure.

The spatial cancer incidence database in Saudi Arabia was designed and developed in the form of an ESRI File Geodatabase on three spatial levels: regional, governorates and cities. Saudi Arabia is divided into thirteen regions; each region is divided into governorates, and each governorate includes a number of cities. The cancer database we obtained had records for individual cancer cases. However, the location of each cancer case was not included. To develop a spatial cancer database, the individual cancer cases were aggregated into city, governorate, regional and national levels. Starting from the city level, all of the cancer cases located in the same city were grouped and aggregated to be represented by that city. Next, all of the cancer cases located in certain cities belonging to a specific governorate were grouped together and represented by that governorate. At the regional level, all of the cancer cases in certain governorates belonging to a specific region were grouped together and represented by that region.

### 2.2. NO_2_ Data

NO_2_ is an omnipresent atmospheric pollutant due to the extensive prevalence of both natural and anthropogenic sources, and is a primarily man-made gas. NO_2_ is produced in the environment as the main emission nitrogen oxides (NOx). The NOx that yield NO_2_ are emitted naturally by biomass burning (e.g., forest fires), lightning, and microbial activity in the soil, while they are emitted due to anthropogenic activities by fossil fuel and biofuel combustion, power plants, heavy industry and vehicular traffic, making it a strong indicator of vehicle emissions. NO_2_ (and other NOx) is a forerunner of a number of harmful secondary air pollutants, including nitric acid and photo oxidants (including ozone) [1,49,50].

**Figure 1:**
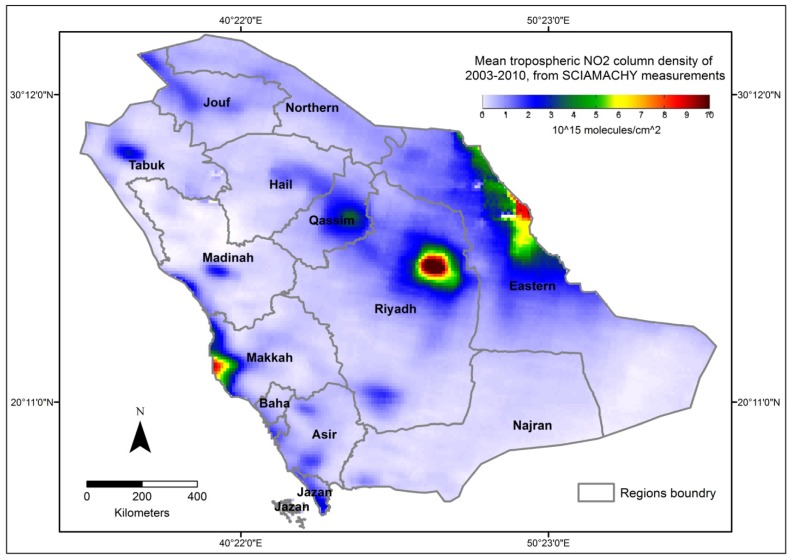
Distribution of mean tropospheric NO_2_ column density, 2003–2010.

The mean tropospheric NO_2_ column density data for cities in Saudi Arabia (Figure 1) were extracted from a global NO_2_ pollution map produced by the Satellite Group in the Max-Planck-Institute for Chemistry in Mainz, Germany [51]. The image shows the global mean tropospheric NO_2_ column density between 2003 and 2010 using Envisat observations as measured by the SCIAMACHY instrument on ESA’s Envisat, the world’s largest satellite for environmental monitoring. “SCIAMACHY is an imaging spectrometer whose primary mission objective is to perform global measurements of trace gases in the troposphere and in the stratosphere. The solar radiation transmitted, backscattered and reflected from the atmosphere is recorded at relatively high resolution (0.2 µm to 0.5 µm) over the range 240 nm to 1700 nm, and in selected regions between 2.0 µm and 2.4 µm. SCIAMACHY has three different viewing geometries: nadir, limb, and sun/moon occultations which yield total column values as well as distribution profiles in the stratosphere and (in some cases) the troposphere for trace gases and aerosols. The nadir and limb viewing strategy of SCIAMACHY yields total column values as well as profiles for trace gases and aerosols in the stratosphere. Additionally, this enables estimates of global trace gas and aerosol content and distribution in the lower stratosphere and troposphere. The measurements obtained from SCIAMACHY enable the investigation of a wide range of phenomena which influence atmospheric chemistry such as measurement in the troposphere: biomass burning, pollution, arctic haze, forest fires, dust storms, industrial plumes; and measurement in the stratosphere: ozone chemistry, volcanic events and solar proton events. The spatial resolution of SCIAMACHY depends on the wavelength region and also on the solar zenith angle. For most NO_2_ measurements, the area is 60 × 30 km^2^. Currently, the analysis is based on a rather limited set of both uncalibrated and calibrated data that have been released by ESA, and therefore has to be considered as preliminary” [50]. A description of the retrieval algorithm used and an application to long-term changes of tropospheric NO2 can be found in Richter *et al*. [52].

Using the global mean tropospheric NO_2_ column density map, we first isolated the area of Saudi Arabia from the global map and then georeferenced the clipped map (Figure 1). The NO_2_ values were first extracted for Saudi cities using the Sample function with the nearest resampling algorithm, and then we aggregated the NO_2_ values at the governorate and regional levels using the Zonal Statistics function in ESRI ArcGIS. An issue associated with the aggregated NO_2_ is the method by which the geographic boundaries of regions and governorates are defined; this difficulty is known as the modifiable areal unit problem (MAUP) [53].

### 2.3. Spatial Statistical Analysis

GWR is a reasonably recent contribution to modeling spatially heterogeneous processes. Using GWR, parameters can be estimated anywhere in the study area given a dependent variable and a set of one or more independent variables measured at areas whose location is known [34,54,55,56,57]. In contrast to the global regression model OLS, GWR can estimate discrete coefficients for each observation, *i.e.*, geographic features. GWR extends the conventional OLS linear regression models that mask significant local variation. The key difference between global and local analyses is that global estimation uses one model for all observations, while GWR estimates a particular local model for each location in space. GWR is capable of generating parameter estimates for every regression point using observations in a given neighborhood. The parameter estimates are characteristically mapped to highlight spatial variation [58]. GWR is an extension from global regression to local regression, with the critical idea that for each regression point *i*, there is a bump of influence around *i* described by the weight function such that sampled observations near *i* have more influence in the estimation of the parameters than observations sampled further away [34]. The GWR model can be expressed as follows:

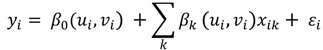
(1)
where the dependent variable *y* is regressed on a set of independent variables, each denoted by *x_k_*, and the parameters are allowed to vary over space. Here, (*u_i_*, *v_i_*) denotes the coordinates of the *i-*th point in space, and *β_k_*(*u_i_*, *v_i_*) is a realization of the continuous function *β_k_*(*u_i_*, *v_i_*) at point *i*; *xi*1, *xi*2, . . ., and *xip* are the explanatory variables at point *i*; and *ε*i are error terms [34,56]. For a given data set, the local parameters *β_k_*(*u_i_*, *v_i_*) are estimated using the weighted least square procedure. The weights *wij* for *j* = 1,.., *n* at each location (*u_i_*, *v_i_*) are obtained as a continuous function of the distance between point *i* and the other data points.

Let:

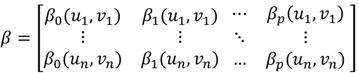
(2)
be the matrix of the local parameters. Each row is estimated by

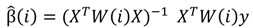
(3)
where *i* = 1, *. . .*, *p* represents the row of the matrix, *X* is the matrix of explanatory variables, *y* is the dependent variable, and *W*(*i*) is an *n* by the *n* spatial weighting matrix of the form:

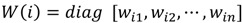
(4)

In global regression models such as OLS, every point has the same weight, whereas in local regression models such as the GWR model, the spatial weight of these points decreases with the distance from the regression point. The weights are computed using a weighting scheme that is known as a kernel. Following the suggestions of Fotheringham *et al*. [34], in this study, the spatial adaptive kernel was applied rather than the fixed kernel because cities are not positioned regularly in the study area, *i.e.*, they are heterogeneous and clustered in some areas. The spatial context is a function of a specified number of neighbors. Where the distribution of cities (in this study) is dense, the spatial context is smaller; where the distribution of cities is sparse, the spatial context is larger. A spatially adaptive kernel is usually formed by sorting the distances of the sample points from the desired regression point *i* and setting the bandwidth so that it includes only the first *N* observations, where the optimal value of *N* is determined by the data. The weight can be computed by using the specified kernel, setting the value of any observation whose distance is greater than the bandwidth to zero and excluding them from the local calibration [57]. Although a number of kernels are possible, the bi-square weighting function is usually used to create adaptive kernels [34] and can be implemented in ESRI ArcGIS [38]. Gilbert and Chakraborty [45] used the bi-square weighting function to produce adaptive kernels for the GWR model that examined the spatial association between cumulative cancer risk from exposure to hazardous air pollutants and explanatory variables such as race, ethnicity and socioeconomic status. Charlton and Fotheringham [57] stated that the bi-square weighting function is a near-Gaussian function with the useful property that the weight is zero at a finite distance and can be expressed as *w_ij_* = [1 − (dij/b)2]^2^, where *d_ij_* is the distance between a calibration point *i* and a sample data point *j* and *b* is the distance to the *N*th nearest neighbor, also known as the bandwidth.

In GWR, the regression model is adjusted based on the data that are geographically close to a specific location. In other words, GWR measures parameters within specified distances (named bandwidths) of each other and weights these parameters from an identified regression reference point using a spatial weight function. The optimal bandwidth distance or the optimal number of neighboring units in the GWR can be specified using either cross-validation or Akaike information criterion (AIC) tests. The AIC is considered the most fitting method for applying the adaptive kernel technique because it considers both goodness-of-fit and degrees of freedom [34,58]. In the present study, the optimal bandwidth size was found by minimizing the AIC value, following previous examples of GWR application [58,59,60,61,62]. The bandwidth was found by minimizing the AIC value. The AIC criterion in GWR is computed as in Hurvich *et al*. [63]:

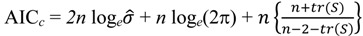
(5)
where *n* is the number of observations in the dataset, 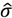 is the estimate of the standard deviation of the residuals, and *tr*(*S*) is the trace of the hat matrix. The AIC can be used to compare models of the same independent variable and compare the global OLS model with a local GWR model [57]. The OLS and GWR models were fitted and mapped using ESRI ArcGIS 10.1.

## 3. Results

Our analysis of the mean tropospheric NO_2_ data for cities in Saudi Arabia (Figure 1) indicates that the high vertical column distributions of NO_2_ were associated with major cities across Saudi Arabia, including Riyadh (central) and Jeddah (western coast), and cities in the Eastern Province, including Dammam, Khobar, Jubail, and Ras Tanura.

**Figure 2:**
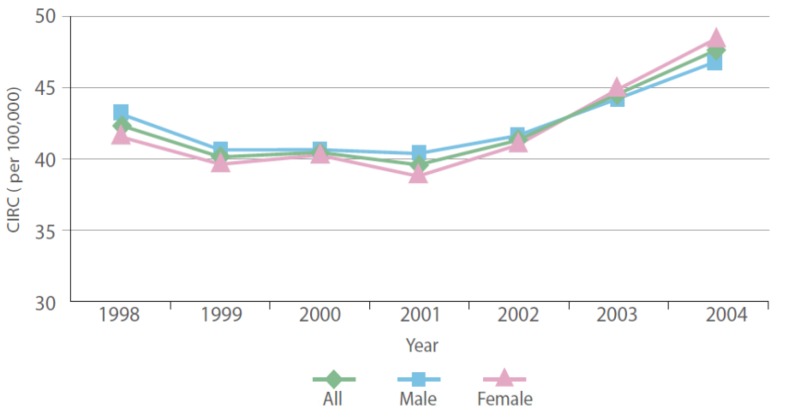
Crude incidence rate (CIR) of all cancers by year.

**Figure 3:**
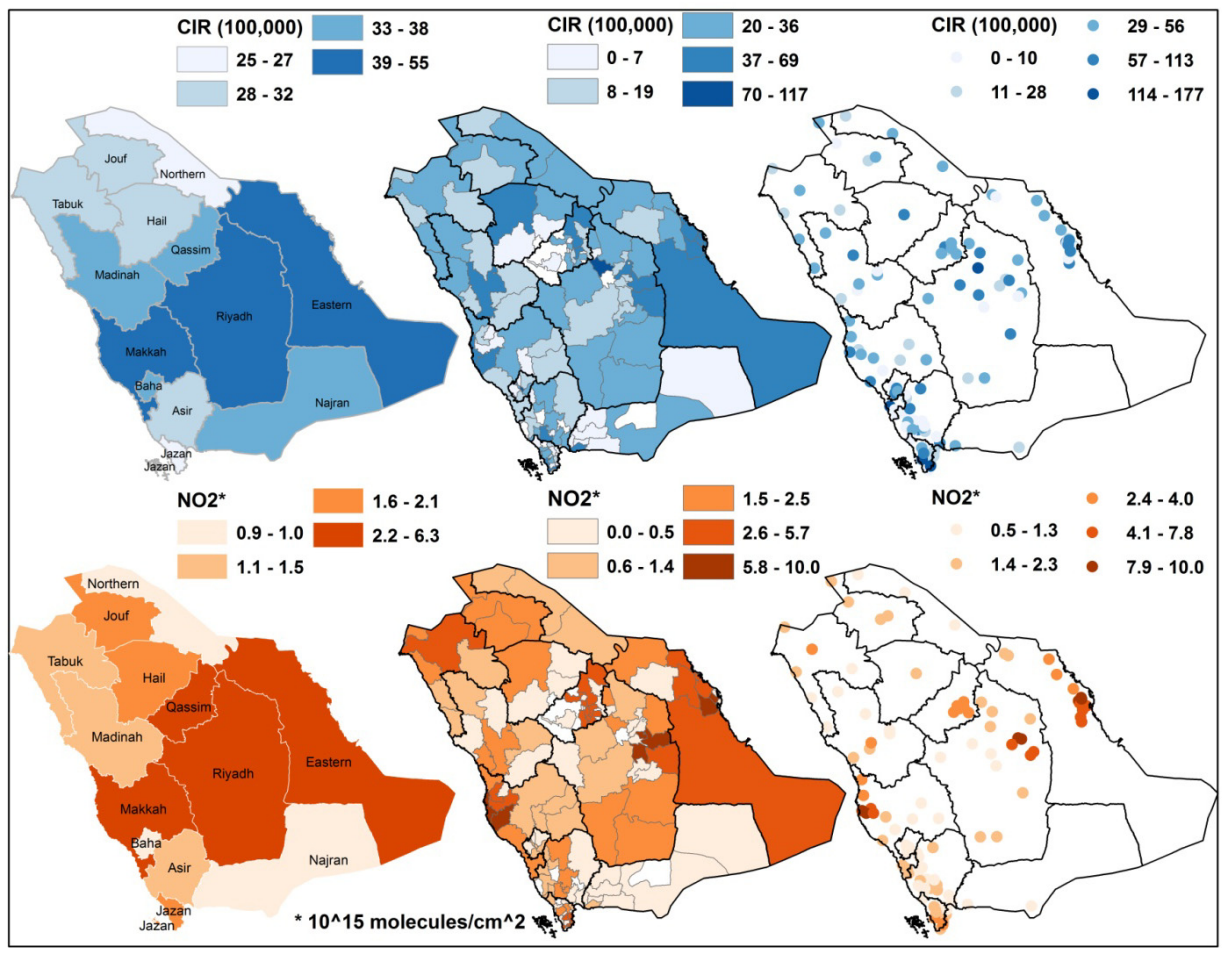
Crude incidence rates of all cancers for the regions, governorates and cities (1998–2004) and mean tropospheric nitrogen dioxide (NO_2_) column density (2003–2010).

A total of 45,532 cancer cases (22,930 males and 22,602 females) were diagnosed among Saudi Nationals between January 1998 and December 2004. In Saudi Arabia, the overall CIR between 1998 and 2004 was 42.41 per 100,000 people in the population (42.61 among males and 42.22 among females) (Figure 2), which indicates that cancer incidence is low among Saudi nationals. In a comparison of the CIRs of overall cancers in the Gulf Cooperation Council (GCC) countries [25], the rate observed among Saudis was lower than that observed in Bahrain, Qatar, Kuwait and Oman (51–93 among males and 47–98 among females) between 1998 and 2001 and lower than the worldwide rate (188 per 100,000) in 2008 [23]. The overall ASR of cancer at all sites in Saudi Arabia during the period between 1998 and 2004 ranged between 70 and 80 per 100,000 people (74–80 among males and 68–80 among females). Therefore, Saudi Arabia exhibited a lower ASR than did other GCC countries, such as Qatar (male: 165.5; female: 172.4) and Bahrain (male: 157.7; female: 144.6) between 1998 and 2001 [25]; the ASR of Saudi Arabia was also lower than the worldwide ASRs of 204 and 165 per 100,000 for males and females, respectively, in 2008 [23]. Liver cancer was the most common, accounting for 8.84% of all cancers in males, followed closely by non-Hodgkin’s lymphoma (NHL) with 8.80% and leukemia with 8.19%; colorectal cancer ranked 4th, followed by lung and prostate cancers. In females, breast cancer was the most common, accounting for 20.2% of all cancers in females, followed by thyroid cancer with 9.3%. Colorectal cancer ranked 3rd and was closely followed by NHL and leukemia. Riyadh region reported 13,063 cancer cases, accounting for 28.69% of all cancer diagnoses between 1998 and 2004, followed by Makkah region, which reported 10,479 cases, accounting for 23.01%, and Eastern province, which reported 7,698 cases, accounting for 16.91%. These three regions showed a significantly increasing trend in the overall number of cancer cases diagnosed between 1998 and 2004. Alahsa governorate (located in the Eastern region) reported the highest CIR, with 284.71 cases per 100,000 population. Ras Tanura governorate (Eastern region) was second, with a CIR of 113.82 cases per 100,000 population, and Shagra governorate ranked third, with a CIR of 110.96 cases per 100,000 population. Baha, Jeddah, Riyadh, Jazan, Dammam and Al-Khobar were among the governorates with the highest rates of all cancers, with CIRs ranging from 53.98 to 69.15 per 100,000 population. Samtah city (Jazan region) reported the highest CIR, with 177.13 per 100,000 population, and Al Qatif city (Eastern region) was second, with a CIR of 173.1 per 100,000 population. Al-Khobar, Shagra, Jazan, Alqunfidhah and Sarat Abidah were among the cities with the highest CIRs of all cancers, which ranged between 135.55 and 171.1 per 100,000 population (Table 1, Figure 2 and Figure 3).

**Table 1:** Number, percentage, crude incidence rate (CIR) and age-standardized incidence rate (ASR) of all cancers, 1998–2004.

Region	All Cases				Males				Females				Avg. NO_2_
	Number	Percent	CIR	ASR	Number	Percent	CIR	ASR	Number	Percent	CIR	ASR	
Riyadh	13,063	28.69	54.88	47.34	6,512	28.4	53.60	46.09	6,551	28.98	56.22	45.37	3.34
Eastern	7,698	16.9	46.42	41.41	3,867	16.86	45.54	43.31	3,830	16.95	47.35	38.53	6.28
Makkah	10,479	23.01	44.51	33.10	5,228	22.8	44.35	31.78	5,251	23.23	44.67	32.00	3.23
Madinah	2,855	6.27	38.57	28.63	1,442	6.29	39.40	28.98	1,413	6.25	37.76	27.72	1.39
Qassim	1,992	4.37	37.45	26.30	1,030	4.49	38.86	27.15	962	4.26	36.06	24.94	3.18
Baha	779	1.71	34.94	18.88	374	1.63	35.95	19.56	405	1.79	34.05	18.25	1.00
Najran	760	1.67	34.19	24.79	419	1.83	37.93	28.56	341	1.51	30.49	20.98	0.90
Hail	948	2.08	32.05	18.68	481	2.1	33.42	18.99	467	2.07	30.74	18.02	1.70
Asir	2,957	6.49	31.09	20.40	1,522	6.64	32.89	20.76	1,435	6.35	29.38	19.53	1.24
Tabuk	1,160	2.55	30.84	27.41	612	2.67	31.63	30.42	548	2.42	30.00	24.93	1.48
Jouf	586	1.29	29.50	21.83	306	1.33	30.75	22.78	280	1.24	28.25	20.71	1.78
Northern	430	0.94	27.55	21.16	210	0.92	26.91	21.63	220	0.97	28.19	21.05	0.93
Jazan	1,613	3.54	25.03	16.77	805	3.51	25.60	16.51	808	3.57	24.49	16.65	2.13

The association between the mean tropospheric NO_2_ and the number and incidence rates of the most common cancers in Saudi Arabia at the region, governorate and city levels were examined using OLS and GWR. A significant association was found, but substantially smaller and less robust associations were also observed. It was found that the number of cancer cases has strong associations with CIR (r^2^ = 0.80, 0.73 and 0.84 for all cases, males and females respectively) and ASR (r^2^ = 0.76, 0.64 and 0.80 for all cases, males and females respectively). This justifies the use of the number of cancer cases in the analysis to detect association between cancer and NO_2_. Table 2, Table 3 and Table 4 show the associations between NO_2_ and the most common cancers at the region, governorate and city levels.

**Table 2:** Association between NO_2_ and the most common cancers at the regional level.

Cancer	OLS-NO	OLS-CIR
All Cancers	0.13	0.43 *
Liver	0.21	0.06
Breast	0.50 *	0.71 *
Colorectal	0.40 *	0.37 *
NHL	0.38 *	0.17
Leukemia	0.40 *	0.12
Thyroid	0.41 *	0.11
Lung	0.62 *	0.59 *
Other Skin	0.32 *	0.00 °
Hodgkin’s Disease	0.55 *	0.17 °
Bladder	0.55 *	0.22 *°
Cervical	0.46 *	0.41 *
Ovarian	0.38 *	0.37 *
Prostate	0.56 *	0.61 *

**OLS**: Ordinary Least Square; **NO**: Number; **CIR**: Crude Incidence Rate; * Statistically significant at *p* < 0.05; ° Violated spatial autocorrelation.

**Table 3:** Association between NO_2_ and the most common cancers at the governorate level.

Cancer	OLS-NO	OLS-CIR	GWR-NO	GWR-CIR
All Cancers	0.31 *°	0.06 *	0.33 °	0.14
Liver	0.26 °	0.05 *	0.26 °	0.10
Brest	0.33 *°	0.09 *°	0.39 °	0.39
Colorectal	0.32 *°	0.06 *	0.34 °	0.14
NHL	0.30 *°	0.03 *	0.33 °	0.06
Leukemia	0.28 *°	0.04 *	0.28 °	0.06 °
Thyroid	0.27 °	0.05 *°	0.27 °	0.18
Lung	0.32 *	0.02 °	0.43	0.44 °
Other Skin	0.28 *°	0.005	0.30 °	0.03
Hodgkin’s Disease	0.30 *°	0.03 °	0.31 °	0.17
Bladder	0.31 *	0.007 °	0.33 °	0.08
Cervical	0.33 *	0.05 *	0.42 °	0.15
Ovarian	0.33 *	0.02	0.39 °	0.05
Prostate	0.31 *°	0.05 °	0.33 °	0.31

**OLS**: Ordinary Least Square; **GWR**: Geographically Weighted Regression; **NO**: Number; **CIR**: Crude Incidence Rate; * Statistically significant at *p* < 0.05 (OLS); ° Violated spatial autocorrelation.

**Table 4:** Association between NO_2_ and the most common cancers at the cities level.

Cancer	OLS-NO	OLS-CIR	GWR-NO	GWR-CIR
All Cancers	0.17 °	0.03 °	0.23	0.17 °
Liver	0.13 °	0.0002	0.17	0.22 °
Brest	0.20 *°	0.10 *°	0.29	0.11 °
Colorectal	0.17 °	0.05 *°	0.24	0.06 °
NHL	0.16 °	0.006 °	0.24	0.14 °
Leukemia	0.14 °	0.006 °	0.18	0.14 °
Thyroid	0.14 °	0.017 °	0.17	0.08 °
Lung	0.23 *	0.11 *°	0.33	0.13 °
Other Skin	0.15 °	0.0003	0.20	0.21 °
Hodgkin’s Disease	0.17	0.005 °	0.22	0.22
Bladder	0.17 *	0.008°	0.25	0.19 °
Cervical	0.18 *°	0.04 *°	0.30	0.05 °
Ovarian	0.18 *°	0.007 °	0.29	0.09 °
Prostate	0.18 *°	0.10 *°	0.24	0.14 °

**OLS**: Ordinary Least Square; **GWR**: Geographically Weighted Regression; **NO**: Number; **CIR**: Crude Incidence Rate; * Statistically significant at *p* < 0.05 (OLS); ° Violated spatial autocorrelation.

At the regional level (Table 2), the OLS method indicated that the numbers of lung, prostate, Hodgkin’s disease, bladder and breast cancers (r^2^ = 0.62, 0.56, 0.55, 0.55 and 0.50, respectively, *p* < 0.05) were significantly positively associated with NO_2_. While using the CIR, the main significant associations were positive associations between NO_2_ and breast, prostate and lung cancers (r^2^ = 0.71, 0.61 and 0.59, respectively, *p* < 0.05). It was found that ASR at the regional level has a stronger association with NO_2_ (r^2^ = 0.51, 0.49 and 0.52 for all cases, males and females respectively) than does CIR (r^2^ = 0.43, 0.37 and 0.47 for all cases, males and females respectively). This implies that if we have data for ASR at finer geographic levels, they might have stronger associations with NO_2_ as well.

At the governorate level (Table 3), the overall values of the coefficient of determination r^2^ were generally less than those found at the regional level. At the spatial governorate level, the OLS method indicated that the numbers of diagnosed breast, lung, bladder, cervical and ovarian cancers (r^2^ = 0.33, 0.32, 0.31, 0.33 and 0.33, *p* < 0.05) were the highest in terms of a significant association with NO_2_. However, the spatial autocorrelation of breast cancer violated the assumption of independence based on the clustered standardized residual error.

The significant associations between NO_2_ and the CIRs of the most common cancers were low among the most common cancers, and the highest was found for colorectal and all cancers (r^2^ = 0.06, *p* < 0.05). Using the GWR method, the highest significant correlation was found between NO_2_ and the number of diagnosed lung cancers (r^2^ = 0.43). The CIR of lung cancer showed the highest correlation (r^2^ = 0.44), but the spatial autocorrelation violated the assumption of independence based on the clustered standardized residual error. A significant correlation between NO_2_ and the CIR of the most common cancers was also found for breast and prostate cancers (r^2^ = 0.39 and 0.31, respectively).

At the cities level (Table 4), the overall values of the coefficient of determination r^2^ were generally lower than those found at the regional and governorate levels. At this spatial level, OLS and GWR were applied for the number of diagnosed cancers and the CIR. Using OLS, the highest significant association with NO_2_ was found for the number of lung cancer diagnoses (r^2^ = 0.23, *p* < 0.05), while for the CIR, there was no significant association (r^2^ ≤ 0.0003, *p* > 0.05). Using GWR, the highest significant correlation was found between NO_2_ and the number of lung cancer diagnoses (r^2^ = 0.33) followed by cervical, ovarian and breast cancers (r^2^ = 0.30, 0.29 and 0.29, respectively). Regarding the CIR, the highest significant correlation was found for Hodgkin’s disease (r^2^ = 0.22), whereas the other most common cancers were violated by the spatial autocorrelation of clustered standardized residual error. Overall, high coefficients of determination (r^2^) were observed in the Eastern, Riyadh and Makkah regions and in their governorates and cities.

## 4. Discussion

This study aimed to investigate whether the number of cases and incidence of the most common cancers in Saudi Arabia between 1998 and 2004 were significantly associated with exposure to NO_2_ urban air pollution using the OLS and GWR models in GIS. This study is the first in Saudi Arabia and the region to use spatial and non-spatial cancer data, the spatial cofounding factor (*i.e.*, distribution surface of NO_2_), and the methods applied.

The high NO_2_ concentrations in the major cities across Saudi Arabia could be attributed to vehicle emissions and the chemical industries. Additionally, the Eastern region contains Saudi Arabia’s massive petroleum resources, as it is home to most of Saudi Arabia’s oil production. The province is also home of the City of Jubail, which hosts the Jubail Industrial City, a global hub for chemical industries and the largest industrial city in the Middle East. It also holds the Middle East’s largest and the world’s fourth largest petrochemical company. The Eastern region also encompasses Ras Tanura city, which is a major oil port and oil operations center for Saudi Aramco, the largest oil company in the world. The NO_2_ concentrations in Riyadh and Jeddah, the two largest cities in Saudi Arabia, could be attributed to the large number of cars and urban activities.

There were statistically significant associations between the concentration of NO_2_ air pollution and the most common cancers diagnosed between 1998 and 2004 in Saudi Arabia. This result can be explained by the fact that NO_2_ is much more concentrated in urban areas, where more cancer cases occur because of the size of the population. However, the coefficient of determination of these associations varied between the spatial levels of analysis (regions, governorates and cities), the methods used (OLS and GWR), the measurement of cancer data employed (diagnosed number or CIR) and the diagnosed cancer sites. Notably, the only results considered in this study were those significant at *p* < 0.05 and the standardized residual errors that were not spatially autocorrelated.

Regarding the spatial level of analysis, the significant coefficients of determination (r^2^) were higher at the regional level (r^2^ =0.32–0.71), weaker at the governorate level (r^2^ =0.03–0.43) and declined slightly at the city level (r^2^ = 0.17–0.33). The finding that the association was higher at the regional level may be attributable to the rural/urban variability in NO_2_, which is fairly visible in Figure 1. However, the low values of the coefficients of determination at the lowest spatial level (*i.e.*, cities) suggest that additional variation remains unexplained. Thus, factors other than NO_2_ may be associated with the risk of cancer.

Robinson [64] coined the terms “ecological fallacy” and “ecological correlation”, which refer to the inappropriate use of an aggregated statistic to make inferences about an individual. This study is considered an ecological correlation because the units of analysis in this study were people within cities, governorates and regions but not individual people: *i.e.*, ecological inferences about the individual were drawn from aggregate data. This is a common concern in ecological studies in which exposure and response are quantified only for aggregates and not individuals [65].

Regarding the methods used, only the OLS method was applied at the regional level because there are thirteen administrative regions in Saudi Arabia, and the minimum recommended number of features to apply GWR is 100. Using OLS, the significant coefficients of determination at the regional level were high (r^2^ = 0.32–0.71). At the governorate and city levels, GWR indicated that the associations between the concentration of NO_2_ air pollution and the most common cancers were marginally improved (r^2^ = 0.03–0.33 using OLS and r^2^ = 0.03–0.43 using GWR for governorates; r^2^ = 0.17–0.23 using OLS and r^2^ = 0.17–0.33 using GWR for cities). Therefore, a non-stationary local model (*i.e.*, GWR) gave a much better account than a global model (*i.e.*, OLS) for spatial estimation and prediction. Although global models mask widespread local variation, local models increase prediction accuracy by offering the opportunity to explore and understand local variations and allowing the spatial drift of regression parameters to be identified, estimated and mapped.

Regarding the employed measurement of cancer data, regardless of the spatial level of analysis or the method used, the significant coefficients of determination were r^2^ = 0.17–0.62 using the number of diagnosed cancer cases, whereas they were r^2^ = 0.05–0.71 using the CIRs. This finding suggests that a correlation exists between NO_2_ and cancer development. A high association between cancers and NO_2_ exposure for both the number and incidence rate might imply that such a relationship is highly focused on urban areas with a high population and high NO_2_ concentration due to urban and industrial activities. This result is largely factual, particularly when one examines the areas with a high association between the two variables. Areas with high associations were clustered in the Eastern and Riyadh regions. The industrial and petrochemical activities in Saudi Arabia are largely located in the Eastern Province, which is the largest producer of oil and related petrochemical activities worldwide as well as a high densely populated area. By contrast, the Riyadh region includes the capital city and is the most populated area in the country.

In terms of tumor location, a high association was observed between the concentration of NO_2_ air pollution and the risk of developing lung and breast cancers, followed by prostate, bladder, cervical and ovarian cancers. This finding corroborates results from other studies. For example, associations have been reported between NO_2_ and lung cancer [4,6,7,8,11], breast cancer [14], bladder cancer [13] and cervical and brain cancers [16].

However, this study is limited because the study cohorts were cancer incidence rates between 1998 and 2004 *versus* the NO_2_ concentration between 2003 and 2010. Exposure must precede the outcome, and a decade may be required for cancer to develop. It would have been preferable to use NO_2_ data for previous decades; unfortunately, such data were not available. One could argue that the overall pattern and trend in the NO_2_ concentration may not have changed substantially. Outdoor NO_2_ air pollution can mainly be attributed to power plants, heavy industrial activities and vehicular traffic. Al-Jeelani [66] stated that there is a lack of data about air pollution generated by power plants in Saudi Arabia and that the most significant source of air pollutants such as NO_2_ is automobiles. The number of automobiles in most Saudi cities increases in tandem with population growth. Heavy industrial activities in Saudi Arabia were established a few decades ago and are concentrated in certain major regions: Eastern, Riyadh and Makkah. Therefore, it can be claimed that the overall pattern and trends related to NO_2_ concentration may not have changed significantly between 1998 and 2004 compared with the period between 2003 and 2010. Moreover, exposure to air pollutants such as NO_2_ is one environmental risk factor for cancer. However, cancer incidence is explained by a combination of genetic, demographic, socio-economic, environmental, behavioral and cultural risk factors. In particular, the variations in cancer incidence are probably associated with many variables, including population aging and growth, tobacco smoking status (intensity and duration), occupational exposures, environmental exposures and factors, dietary habits (including unhealthy dietary habits), physical inactivity, the prevalence of obesity, genetic factors, the lack of screening programs and the accessibility of specialized cancer centers [22,23,24,25,26,27,28,29,30,31,32]. Regrettably, there appears to be a lack of data on these covariates in Saudi Arabia, and thus, they could not be analyzed in the present study.

## 5. Conclusions

This study is the first of its kind in Saudi Arabia because it relied on reliable cancer data acquired from the Saudi Cancer Registry, the spatial database of cancer incidence rates developed by the authors and the global NO_2_ map created using the Envisat observations, as measured by the SCIAMACHY instrument on ESA’s Envisat. Additionally, the statistical methodology employed in this study was a combination of global models, such as OLS, and local spatial statistical models, such as GWR, which captured and explained both the global and local heterogeneity and variations in the number of cancer cases and incidence rates. However, there is a lack of information on other contributing (cofounding) factors. Although an association was found between exposure to NO_2_ air pollution and the development of some cancers, these inferences may be inaccurate to a certain extent because they are uncertainly supported by the aggregate data. If exposure to NO_2_ was found in individual-level data, the inferences would be more reliable and could be used strategically to create health policies, health planning services and preventive policies and to control emissions. Environmental, demographic, behavioral, socio-economic, genetic and other risk factors are of great importance in spatial epidemiological studies of cancer. Countries with noticeable industrial expansions and increased burden of cancer such as Saudi Arabia should establish a nationwide spatial database of risk factors at the individual level. Such data will be vital for spatial epidemiological studies and for studies related to more general health concerns.
